# Water quality assessment based on the water quality index method in Lake Poyang: The largest freshwater lake in China

**DOI:** 10.1038/s41598-017-18285-y

**Published:** 2017-12-21

**Authors:** Zhaoshi Wu, Dawen Zhang, Yongjiu Cai, Xiaolong Wang, Lu Zhang, Yuwei Chen

**Affiliations:** 1 State Key Laboratory of Lake Science and Environment, Nanjing Institute of Geography and Limnology, Chinese Academy of Sciences, 73 East Beijing Road, Nanjing, 210008 China; 2 Institute for Quality & Safety and Standards of Agricultural Products Research, Jiangxi Academy of Agricultural Sciences, Nanchang, 330200 China

## Abstract

Twenty-four samplings were conducted every 3 months at 15 sites from January 2009 to October 2014 in Lake Poyang, and 20 parameters were analyzed and classified into three groups (toxic metals, easily treated parameters, and others). The assessment results based on water quality index (WQI) showed that the water quality in Lake Poyang was generally “moderate”, according to the classification of the surface water quality standard (GB3838-2002) in China, but a deteriorating trend was observed at the interannual scale. Seasonally, the water quality was best in summer and worst in winter. Easily treated parameters generally determined the WQI value in the assessment, especially total nitrogen (TN) and total phosphorus (TP), while toxic metals and other parameters in Lake Poyang were generally at low and safe levels for drinking water. Water level (WL) has a net positive effect on water quality in Lake Poyang through dilution of environmental parameters, which in practice means TN. Consequently, local management agencies should pay more attention to nutrient concentrations during the monitoring schedule, as well as during the low-water periods which manifest a relatively bad water quality state, especially with the prevailing low WL observed recently in Lake Poyang.

## Introduction

Surface water quality deterioration has become a serious concern worldwide due to increased pollution and climate change^1–3^. Such deterioration threatens the use of water resources, especially the drinking water supply, and economic development. However, according to UNICEF^4^, improving the water supply remains a challenge, especially in Asia. Poor water quality has been linked to public health concerns, mainly through the transmission of water-borne diseases. Therefore, many countries have implemented water quality protection measures and monitoring regimens^5–7^. Furthermore, to better understand water resource conditions, it is critical to assess water quality, especially the major contributors to its spatial and temporal variations.

Many studies have established various methods to assess water quality, including multivariate statistical methods^8^, modeling techniques^9^, and methods based on multi-metric indices^10^. The water quality index (WQI) was designed by Horton^11^ and Brown^12^ and has been further developed by various researchers^13–15^. Although various formulas are available to calculate the WQI, all of them effectively convert numerous physical and chemical parameters into a single value that reflects the water quality level, thus eliminating differences between the parameters used individually in the assessment. The WQI method has been widely applied to evaluate both surface water and groundwater quality. Hou^16^ evaluated the spatial and temporal variations in the water quality of typical reservoirs on the Yellow River, China, and analyzed the major contaminants based on the WQI. Using a method that combined the WQI and GIS, Sener^17^ characterized the spatial differences in water quality in the Aksu River, Turkey. Based on a WQI assessment, Selvam^18^ argued that the groundwater quality around the coastal city of Tuticorin in southern India was impaired by anthropogenic factors.

In China, more than 2,600 lakes have surface areas >1 km^2^ and have been subjected to high nutrient loads due to economic development, population growth, and urbanization^19–21^. Consequently, the water quality in these lakes has deteriorated, and the associated levels of biodiversity and functionality have been diminished, which further threatens water security^21^. Similar to protection programs in other countries, the Chinese government has developed a series of measures to control the decline in freshwater quality. One such program is the “Major Science and Technology Program for Water Pollution and Treatment (2006–2020)”, which recognizes water pollution as a national concern. These programs have generally been implemented in areas with severe eutrophication, such as Lake Taihu, Lake Chaohu, and Lake Dianchi. However, little attention has been given to lakes at risk of or with unobserved eutrophication.

Lake Poyang is the largest freshwater lake in China. The lake is an important source of drinking water as well as for water for irrigation, aquaculture and industry. Meanwhile, the water quality of Lake Poyang is heavily affected by dredging, transportation, tourism, and other factors. Obviously, the water quality of Lake Poyang is important for human uses, especially as a source of drinking water. However, cyanobacteria blooms were recently observed by Wu *et al*.^22^. Consequently, multiple studies of the water quality of Lake Poyang have been performed, including analyses of the distribution of and variation in water quality parameters^23–25^ and potential pollution sources^26^. Additionally, while the surface water quality in Lake Poyang has been previously evaluated, the assessment results have primarily been based on single-factor assessments^27,28^, in which assessment parameters were individually evaluated and water quality was determined according to the most impaired parameter. However, water quality assessments in other areas have adopted more comprehensive methods that simultaneously consider multiple water quality parameters rather than focusing on the most impaired parameter^14,17,29^. In addition, the results based on different assessment methods may cause confusion in understanding the water quality and resource uses of Lake Poyang. Therefore, a more reliable method is necessary to determine the water quality of the lake. Such a method must overcome the limitations of conventional approaches and provide a foundation for water resource protection.

Water level fluctuation (WLF) in shallow lakes is largely affected by regional climatic conditions and human activities^30,31^. Because Lake Poyang is connected to the Yangtze River, its water level (WL) fluctuates considerably, particularly at the seasonal scale^23^, depending on the balance between the Yangtze River and Lake Poyang as well as local precipitation^32,33^. Recently, the persistent dryness of Lake Poyang has attracted the attention of local governments and researchers because it has caused water supply and irrigation crises for 12.4 million inhabitants^34,35^. Therefore, WL is an important parameter in the management of water resources by local government. However, knowledge about the effect of WLFs on the water quality of Lake Poyang remains limited. Hence, it is essential to determine the effect of WL on evaluation results, which can improve water quality management in Lake Poyang.

Our study was based on a large data set of 20 parameters measured at 15 sampling sites four times each year over a 6-year period. The WQI method was performed to assess the water quality of Lake Poyang. The 20 parameters were divided into 3 groups: Group 1 (toxic metals), Group 2 (easily treated parameters by a sewage treatment plant), and Group 3 (other parameters). The WQI values for each group were calculated, i.e., W(1) for toxic metals, W(2) for easily treated parameters, and W(3) for other parameters. The final WQI value for all three groups was determined by the maximum value among W(1), W(2), and W(3). The primary objectives were as follows: (1) to illustrate the water quality conditions and spatial and temporal variations in Lake Poyang, (2) to explore the key contributing parameters in determining water quality, and (3) to examine the water quality performance of a Yangtze-connected lake with characteristically high WLF. Our study provides a comprehensive understanding of the water quality evaluation in Lake Poyang. Such an understanding is crucial for water resource management, and it also enhances the knowledge base underlying water quality assessments of river-connected lakes worldwide.

## Results

### Water quality assessment based on WQI

**Figure 1:**
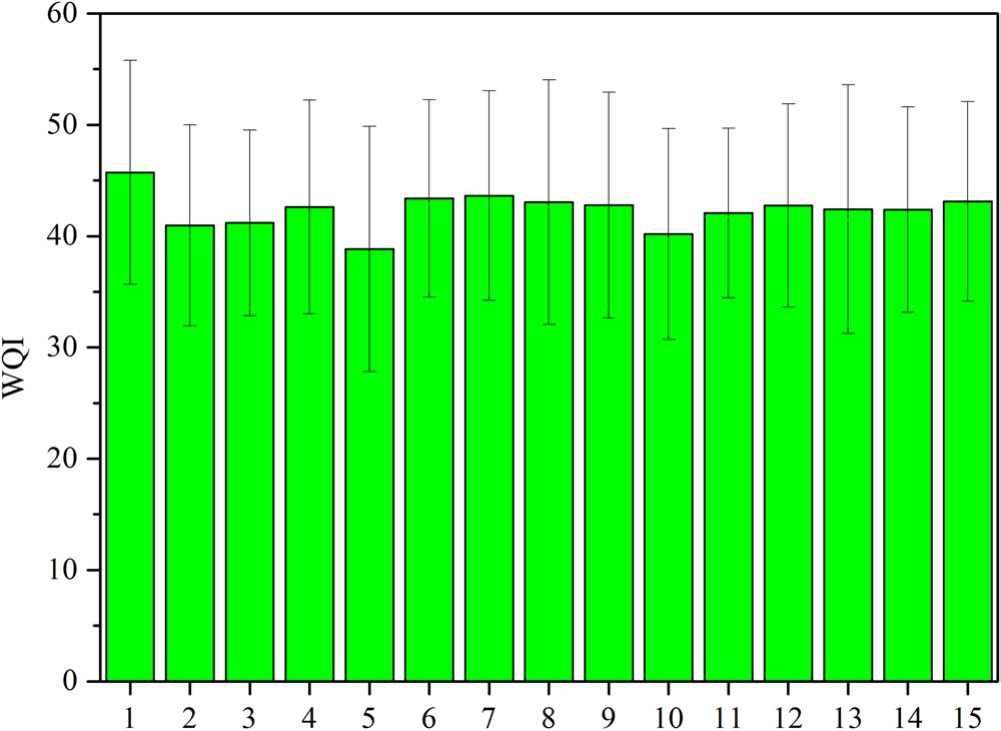
Spatial distribution of water quality index (WQI) values with in Lake Poyang from 2009–2014. Results were expressed as mean ± standard deviation (SD) based on WQI values.

From 2009 to 2014, the average WQI value in Lake Poyang was 42.34 ± 9.98 (standard deviation) based on all WQI calculations. According to the water quality classification standard, the water quality was rated “moderate” and the mean value was close to the “good” threshold. Only 1 observation had a WQI value below 20, i.e., “excellent” water quality overall. According to the WQI classification levels, “moderate” and “good” were the main states in water quality of Lake Poyang, accounting for 49.17% and 43.61% of all the samplings, respectively. Spatially, the highest mean WQI was detected at Site 1 (45.72), while the lowest (38.84) was at Site 5, which was the only site with a WQI value below 40, indicating the “good” water quality (Fig. 1).

**Figure 2:**
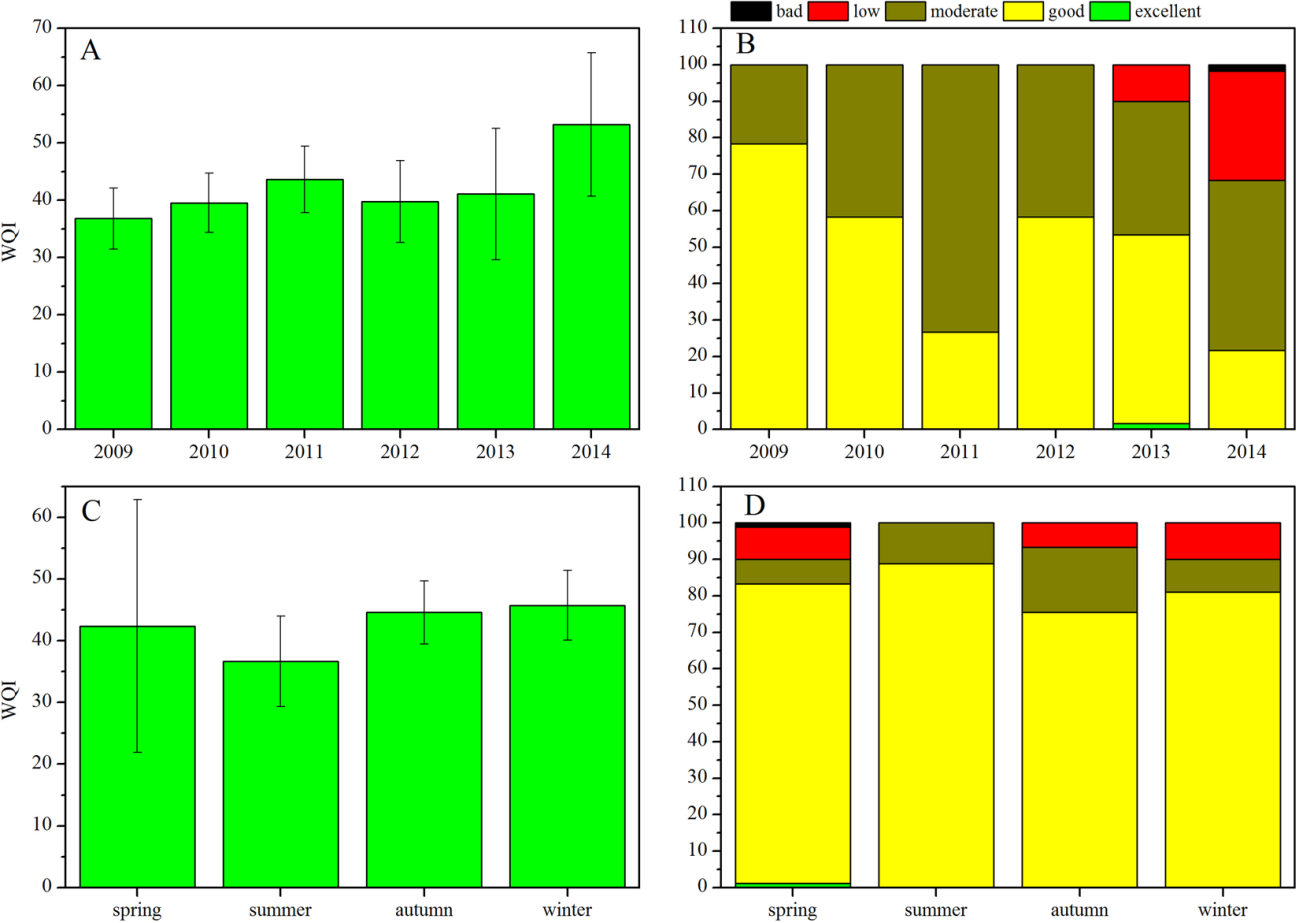
Temporal distribution of WQI values and relative composition of water quality grade in Lake Poyang from 2009–2014. Results were expressed as mean ± SD based on WQI values.

At the interannual scale, the mean WQI values were below 40 in 2009, 2010, and 2012, with values of 36.80, 39.54, and 39.76, respectively (Fig. 2A); however, in 2011, 2013, and 2014, the mean values were higher, especially in 2014, with a value of 53.22 and a relatively high percentage of “bad” and “low” states (Fig. 2B). Overall, the WQI increased from 2009 to 2011, following the variation of the percentage of “moderate” (which was opposite to the percentage of “good”). A similar phenomenon was observed from 2012–2014, and the “bad” and “low” states were mainly observed in 2013 and 2014. Therefore, according to our 6-year study, the water quality in Lake Poyang was “good” or “moderate” for equal amounts of time, but the general trend of water quality was deteriorating, and “bad” and “low” water quality states were detected more recently.

Seasonally, variations also existed in WQI values (Fig. 2C). The average WQI value in summer was 36.66, lower than the values in the other three seasons and the highest value was observed in winter (45.73). Water quality states worse than “moderate” were observed in spring, autumn, and winter, but not in summer (Fig. 2D). Thus, the water quality of Lake Poyang was best in summer (i.e., during the high-water period) achieving a “good” water quality rating, while the water quality was rated “moderate” in the other seasons and worse in winter.

### Major contributors to the WQI

Among the three types of water quality parameters, easily treated parameters played a predominant role in the WQI. The WQI value of easily treated parameters (W(2)) was determined by the concentrations of pH, dissolved oxygen (DO), total nitrogen (TN), total phosphorus (TP), ammonium (NH_4_-N), and permanganate index (COD_Mn_). On one hand, the average W(2) was 41.37 based on all the data and nearly equivalent to the average WQI (42.34) in Lake Poyang. The mean WQI value of toxic metals and other parameters (i.e., W(1) and W(3)) were 5.71 and 10.43, respectively; both were much lower than the W(2) value and stayed at a relatively low level at temporal (seasonal and interannual) scales (Table 1). Specifically, the mean WQI values of arsenic (As), mercury (Hg), cadmium (Cd), chromium (Cr), and lead (Pb), which were classified as toxic metals in our study, were 3.37, 8.40, 2.67, 2.26, and 3.95, respectively. Regarding the other parameters (i.e., copper (Cu), zinc (Zn), iron (Fe), manganese (Mn), cobalt (Co), nickel (Ni), vanadium (V), chloride (Cl), and sulphate (SO_4_) in Group 3), the average WQI value of each parameter was no higher than 18.75, and all were within the threshold of excellent water quality. The detailed mean concentration and range of each parameter mentioned above can be found in the supplementary material (Table S1). On the other hand, 96.11% of the final WQI values were determined by the W(2), while W(1) and W(3) determined only 0.56% and 3.33%, respectively. Furthermore, the correlation coefficient between WQI and W(2) was 0.91 (*p* < 0.01), much closer than that between WQI and W(1)/W(3), which were 0.14 (*p* = 0.041) and 0.42 (*p* < 0.01), respectively.

**Table 1:** Variations in water quality index (WQI) values among the three groups and water level (WL), summarized as mean and range in Lake Poyang from 2009 to 2014 (WL in m).

		W (1)	W (2)	W (3)	WL
general		5.71 (0.00–81.11)	41.37 (7.03–78.22)	10.43 (1.29–81.74)	12.26 (7.74–19.35)
seasonal	spring	3.00 (0.00–63.94)	45.47 (24.34–78.22)	9.29 (2.20–33.82)	11.09 (9.10–13.45)
	summer	10.67 (0.00–81.11)	38.76 (7.03–53.76)	16.10 (2.74–81.74)	17.18 (15.48–19.35)
	autumn	3.79 (0.00–26.35)	36.66 (27.40–53.15)	7.22 (1.19–33.36)	12.07 (9.49–15.09)
	winter	2.51 (0.00–29.83)	44.59 (25.79–69.05)	7.66 (1.67–34.51)	8.71 (7.74–11.36)
interannual	2009	2.27 (0.00–24.57)	36.80 (25.79–50.72)	6.83 (1.53–19.95)	11.18 (7.95–16.40)
	2010	0.48 (0.00–28.77)	39.54 (29.11–59.63)	12.23 (4.00–33.82)	13.44 (7.96–19.35)
	2011	3.21 (1.00–13.94)	43.64 (30.53–54.48)	6.76 (1.19–17.83)	11.07 (9.10–15.59)
	2012	4.44 (0.00–23.74)	39.76 (27.40–56.58)	6.12 (1.78–34.51)	12.23 (7.83–17.67)
	2013	5.39 (0.36–63.94)	40.69 (7.03–69.05)	6.85 (2.89–26.72)	13.06 (10.69–16.49)
	2014	12.66 (0.00–81.11)	47.79 (29.43–78.22)	27.83 (1.67–81.74)	12.60 (7.74–18.54)

More specifically, nutrient species, especially TN and TP, accounted for the high WQI values of Group 2. The average WQI value of TN (I_TN_) was 83.06, more than twice that of W(2) (Table 2). The mean WQI value of TP (I_TP_) was 70.21, which also led to high WQI values in Group 2. Furthermore, the results of a correction analysis showed that TN was the parameter most correlated with the W(2) and the WQI, with Pearson correlation coefficients of 0.60 and 0.55, respectively (Table 2). TP also played an important role in the evaluation; its correlation coefficient with WQI was 0.50. Hence, easily treated parameters generally determined the WQI value in the assessment, especially those of nutrient species (TN and TP), while toxic metals and other parameters were generally at low and safe levels in Lake Poyang for drinking water.

**Table 2:** Distribution of easily treated parameters after normalization and their Pearson correlation relationships with W(2)/WQI.

Parameter	Mean (range)	coefficient		n
		WQI (2)	WQI	
TN	83.06 (34.11–100.00)	0.60**	0.55**	359
TP	70.21 (29.33–100.00)	0.36**	0.50**	356
NH_4_-N	26.45 (2.27–100.00)	0.51**	0.39**	299
COD_Mn_	28.80 (3.54–92.04)	0.54**	0.39**	360
pH	10.83 (0.00–100.00)	0.39**	0.31**	360
DO	26.38 (6.64–99.80)	−0.13*	−0.093	344

^**^ *p* < 0.01; ^*^0.01 < *p* < 0.05.

**Figure 3:**
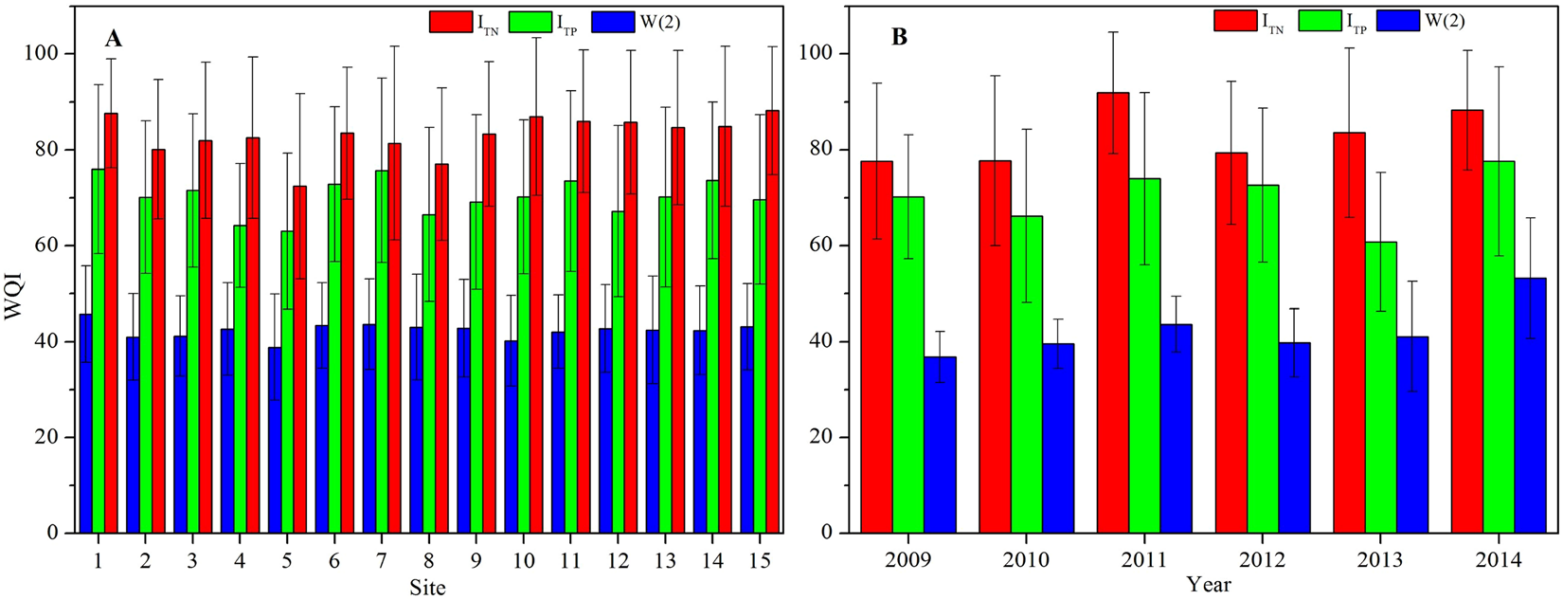
Spatial (**A**) and interannual (**B**) variations in the water quality index (WQI) values of TN, TP, and G2 in Lake Poyang from 2009–2014. Results were expressed as mean ± SD based on WQI values.

Spatially, the I_TN_ values were all higher than the I_TP_ values at all 15 sites, and both I_TN_ and I_TP_ were much higher than the W(2) values (Fig. 3A). The mean I_TN_ at the sampling sites ranged from 72.42 to 88.15 with the maximum at Site 15, which was near the Yangtze River. The minimum mean I_TN_ value was observed at Site 5, as was the minimum I_TP_ value. Similar to the WQI, the maximum value of I_TP_ was found at Site 1. Spatially, there were no obvious differences in I_TN_, I_TP_ and W(2), which was also similar to the distribution of the WQI.

At the interannual scale, the average I_TN_ ranged from 77.62–91.88 during the 2009–2014 period, and the mean I_TN_ value was at least 38 points higher than W(2) in each year (Fig. 3B). The I_TN_ exhibited wide variation over the 6 years of the study. In 2011, the average I_TN_ was extremely high, followed by the average I_TN_ in 2014. The average I_TP_ value in 2013 was 59.11, which was lower than that in the other years. Additionally, the maximum mean I_TP_ of 77.57 was observed in 2014. Therefore, the gap between I_TP_ and the corresponding W(2) narrowed by at least 18 points. The average value of W(2) was higher in 2014 and was also elevated in 2011 (Fig. 3B).

### Relationships between WL and WQI/typical variables

The WL exhibited wide variation across the four seasons in Lake Poyang (Table 1). The highest mean level (17.18 m) appeared in summer and had a range of 15.48–19.35 m that was extremely different from those in the other three seasons. The mean WL in winter was 8.71 m, much lower than that in the other three seasons. No obvious difference in WL was observed between spring and autumn; the mean values were 11.09 m and 12.07 m, respectively. The annual WLF was large, with a mean value of 8.80 m during our study period. The WLF in 2010 was the largest, at 11.39 m, while it was much narrower (5.80 m) in 2013. In terms of the interannual variations, the mean WL varied in a narrow range from 11.07–13.44 m, and the maximum and minimum values appeared in 2010 and 2011, respectively.

**Figure 4:**
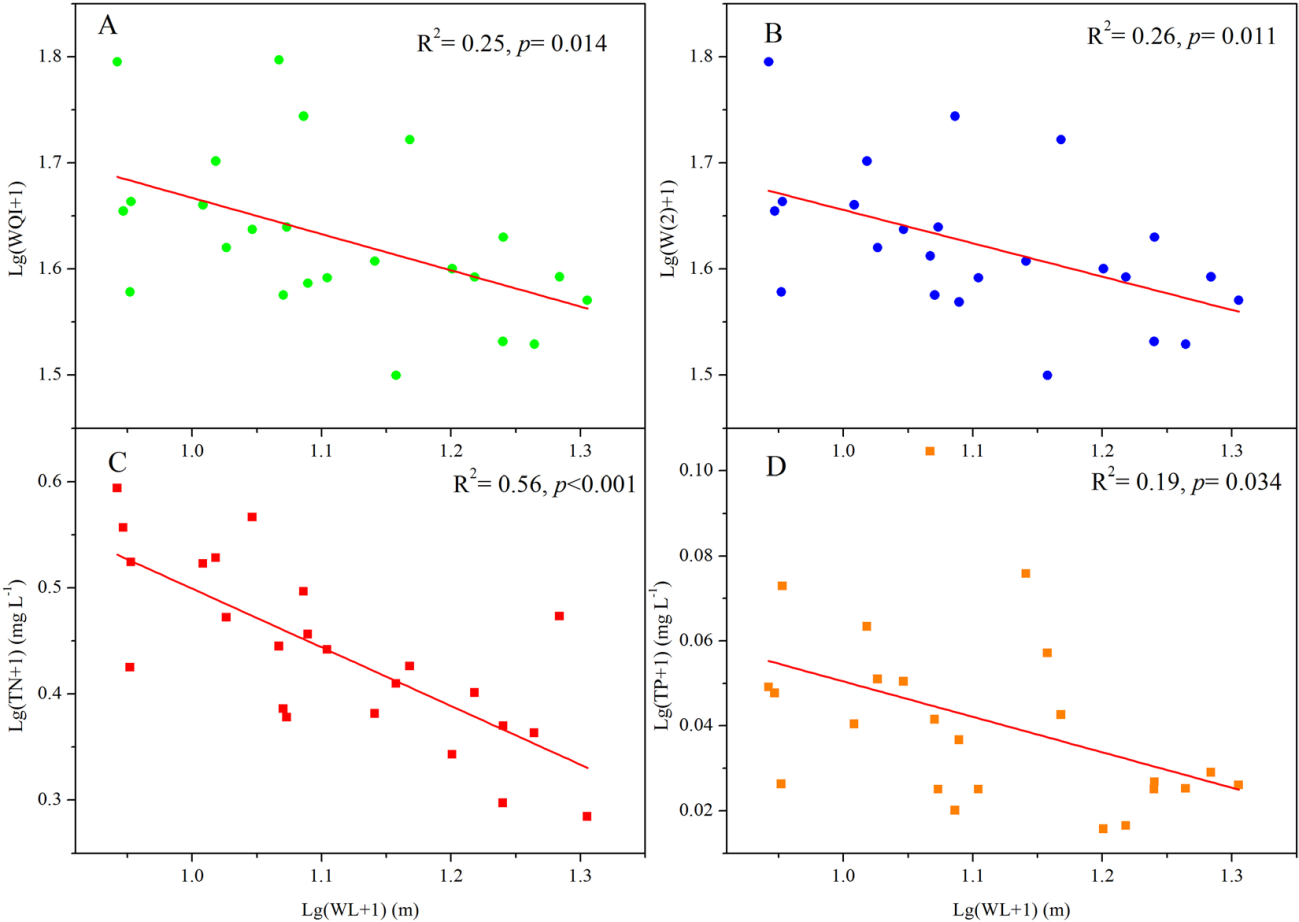
The effects of water level (WL) on WQI, W(2), TN, and TP at the seasonal scale in Lake Poyang from 2009 to 2014.

The results of a Pearson correlation analysis showed that the WL was significantly and negatively correlated with the WQI at the seasonal scale (*R* ^2^ = 0.25, *p* = 0.014, n = 24) (Fig. 4A). The correlations between W(1), W(2), W(3) and WL were also evaluated. WL had a significant and negative effect on W(2) (Fig. 4B). However, no significant relationship was observed between WL and W(1) or between WL and W(3), with *p* values of 0.975 and 0.629, respectively. Specifically, of the six selected parameters that influenced W(2), TN was significantly (*p* < 0.001) and inversely related to WL with an *R* ^2^ of 0.56 (Fig. 4C). Additionally, TP exhibited a close relationship with WL (*R* ^2^ = 0.19, *p* = 0.034, n = 24) (Fig. 4D), but no significant correlation was detected between WL and pH, NH_4_-N, or COD_Mn_ values, which had *p* values of 0.70, 0.21, and 0.091, respectively. To assess the relationship between WL and WQI at the interannual scale, separate WL-WQI regressions were performed for spring, summer, autumn, and winter. The correlation coefficients were negative in all seasons, indicating the negative effect of WL on WQI at the interannual scale. However, all *p* values exceeded 0.05, which may be related to the lack of significant variations in WL from 2009–2014.

## Discussion

### Water quality conditions

According to the WQI method, the overall water quality in Lake Poyang was “moderate”, and the mean value was close to the “good” threshold. These results indicated that the water quality of Lake Poyang still met Chinese drinking water requirements during the study period, but that it was deteriorating at the interannual scale and varied seasonally. Although there are numerous methods for assessing water quality, the water quality condition of Lake Poyang is still poorly understood. Wang^36^ noted that the water quality of Lake Poyang was “poor” from 2010–2012 based on a single-factor method, which was worse than our result. However, Liu *et al*.^37^ argued that the water quality of Lake Poyang generally met the “moderate” requirement from 2008–2012 based on T, pH, DO, TP, NH_4_-N, and COD_Mn_ values (i.e., the easily treated parameters in our study). Therefore, the overall water quality state, i.e., “moderate” or “poor”, varied considerably, and this state reflected whether the water was suitable to drink. Single-factor methods have been the most commonly used in the water quality assessments of Lake Poyang. Although single-factor methods provide valuable information (e.g., the major parameter that influence water quality), the results based on the most-impaired parameter cannot effectively reflect the water quality state. Instead, comprehensive methods based on multiple parameters can benefit water quality evaluation, and such methods have been increasingly adopted in various studies^17,38^. Our 6-year study adopted the WQI method and considered 20 parameters including toxic metals, easily treated parameters, and other parameters. Hence, the results of our study, which was conducted at more comprehensive scale, form a more acceptable water quality assessment of Lake Poyang.

Spatially, narrow variations in WQI were observed among all 15 sampling sites. This result was likely due to water mobility in Lake Poyang. Because Lake Poyang is connected to the Yangtze River, the water retention time of the lake is approximately 10 days, and this retention time is shorter during the dry season^23,32^. Thus, the high mobility and rapid exchange reduce the spatial differences in water quality. Additionally, the water quality of the inflows to Lake Poyang may have affected the spatial variations in water quality on a certain level. The maximum and minimum WQI values were observed at Sites 1 and 5. Site 1 was located in the southern part of Lake Poyang, which receives large amounts of nutrients and organic pollution from inflows, especially the Gan River, while Site 5 was mainly affected by the Xiu River. Based on our data from monitoring the inflows, the contaminant concentrations are relatively high in the Gan River but low in the Xiu River; the average concentration of TN, TP, and NH_4_-N was 2.26 mg L^−1^, 0.14 mg L^−1^, and 0.71 mg L^−1^ in Gan River from 2009 to 2014, respectively, while the corresponding values in the Xiu River were 1.39 mg L^−1^, 0.08 mg L^−1^, and 0.24 mg L^−1^. These trends were consistent with the findings of Lv^39^. With respect to temporal variation, we found a deteriorating trend at the interannual scale and the best water quality was observed in summer. The temporal variations in the water quality of Lake Poyang were relatively consistent based on various assessment methods. Based on the average assessment result of NH_4_-N, TP, and COD_Mn_, Liu *et al*.^37^ reported a declining interannual trend in water quality. Additionally, Zhang *et al*.^40^ used eutrophication and ecological indicators to assess the ecosystem conditions in Lake Poyang and found that the relative ecological status of the lake was best in summer, second best in autumn, and it deteriorated in winter, a result similar to ours.

### The dominant role of easily treated parameters

Easily treated parameters dominated the evaluation result of the water quality in Lake Poyang, while toxic metals and other parameters were generally at low and safe levels for drinking water. According to the WQI calculation in our study, the WQI valued of easily treated parameters, i.e., pH, DO, TN, TP, NH_4_-N, and COD_Mn_, generally determined the final WQI values in Lake Poyang; particularly, the nutrient species (TN and TP) played a crucial role, with obvious higher values than W(2). Our result was consistent with Wang^36^, who found that nutrients were the primary parameters influencing the water quality of Lake Poyang, while other parameters included in the surface water quality standard (GB3838-2002) in China were in a “good” state. Regardless of TN, Liu *et al*.^37^ suggested that TP determined the water quality in Lake Poyang. The WQI values were also consistent with the seasonal and interannual variation of nutrient concentrations in Lake Poyang^23,24^. Interannually, the TN and TP concentrations increased from 1.51 mg L^−1^ to 2.09 mg L^−1^ and 0.098 mg L^−1^ to 0.14 mg L^−1^, respectively, during the period of 2009–2014. Thus, these increases in TN and TP were the major contributors to the deteriorating water quality trend at the interannual scale. Additionally, the mean concentrations of TN and TP were both higher in winter and lower in summer, which were in accordance with the seasonal variations of WQI in Lake Poyang.

Nutrient pollution is a common problem in aquatic ecosystems. Based on our data from monitoring the other four largest freshwater lakes in China (i.e., Lake Dongting, Lake Taihu, Lake Hongze, and Lake Chaohu), the TN concentrations all exceeded 1.80 mg L^−1^ during the period of 2009–2015, and the TP concentrations were larger than 0.1 mg L^−1^ in Lake Taihu, Lake Hongze, and Lake Chaohu. Additionally, Dou and Jiang^23^ suggested that TP was the major parameter affecting the water quality in these five lakes in 1980s, based on collected data for pH, DO, TN, TP, NH_4_-N, nitrate, nitrite, COD_Mn_, As, Hg, Cd, Cr, Pb, Cu, Zn, Fe, Cl, SO_4_, cyanide, petroleum, volatile phenol, biochemical oxygen demand, and total coliforms. A threat to water quality from nutrient loading was also observed in other areas^41–43^. With regard to heavy metals, Zhang *et al*.^25^ investigated their distributions in Lake Poyang in 2011 using the same 15 sampling sites used in this study. The result of Zhang’s study suggested that toxic metals (As, Cd, Pb) and other metals (Cu, Zn, Co, Fe, Mn, Ni, and V) were not the primary parameters influencing the water quality of Lake Poyang and that they all met at least the “good” threshold for drinking water quality in China^25^. Hence, nutrients are likely the key parameters that determine the water quality of Lake Poyang.

### The effect of WL on water quality

WL affected the water quality in Lake Poyang, primarily by dilution of nutrient concentrations at a seasonal scale. Based on the WQI method, our study shows that increased WL has a net positive effect on the water quality of Lake Poyang, especially at the seasonal scale. Recent research has shown that WL is an important parameter regulating the structure and function of natural lake ecosystems^44^. Moreover, because Yangtze-connected lakes have high WLFs, WL is an important parameter affecting their limnological characteristics^23,45,46^. As one of the two lakes still connected to the Yangtze River, Lake Poyang is characterized by large WLF, which has been shown to be the important parameter influencing water retention time, phytoplankton growth, and energy flows ^23,47^. In our study, inverse relationships were observed between WL and the WQI values, as well as W(2)–especially TN, which was a major contributor to the WQI in Lake Poyang. Similar phenomenon occurred in Lake Dongting, the other Yangtze-connected lake in China. According to 15 years of data, the concentrations of TN and COD_Mn_ increased with reduced WL^46^. On one hand, dilution from precipitation likely plays an important role in this inverse relationship in Lake Poyang. Wu *et al*.^24^ demonstrated that variations in seasonal nutrient concentrations were likely affected by the dilution effects of changes in WL, and Liu *et al*.^37^ found that the water quality of inflows was better at high WLs than at low WLs, which was consistent with the results of Wu *et al*.^23^. On the other hand, water flow can induce sediment resuspension and nutrient release^48^ that can cause water quality to deteriorate over time. The effect of water flow on resuspension is high in winter and low in summer in Lake Poyang, which may be affected by the seasonal variations in water level^27^.

A positive effect of high WL on water quality has also been found in other areas. Based on a modeling approach, Hakanson^49^ reported that a WL reduction of approximately 4–5 m from the maximum WL caused water quality deterioration in Lake Kinneret, which is a subtropical lake like Lake Poyang. Because of the extreme decrease in WL (i.e., from approximately 525 m (above sea level) at the beginning of the 1980s to approximately 510 m during the 1990s), the trophic state of Lake Vegoritis changed from oligotrophic at the beginning of the 1980s to mesotrophic during the 1990s^50,51^. Regardless of the trophic state, a general decrease in water quality has been observed in Mediterranean aquatic ecosystems during the low-water period^52^.

### Implication for environmental management

Our study provided a picture of water quality on a large scale using WQI method and established the principal parameters affecting water quality in Lake Poyang. The result will benefit future assessments of water quality in Lake Poyang as well as local management agencies. First, WQI plays an important role in water quality assessment, because it combines several environmental parameters and effectively converts them into a single value that reflects the water quality condition. In contrast, when using a single parameter for water quality assessment, managers will likely receive different results of water bodies based on the different parameters measured (e.g., TN, TP, COD_Mn_, NH4-N). Thus, managers are unclear about using which parameter to evaluate and what the overall state of water bodies is. Additionally, water quality determined by the most impaired parameter obviously cannot reflect the true water quality state. As an integrated indicator value, WQI is crucial for managers, who need concise information about water quality rather than various assessment results by different methods. Second, as noted above, easily treated variables, particularly nutrients, determine the water quality condition in Lake Poyang. Therefore, to improve the water quality in Lake Poyang, local management agencies should pay more attention to the nutrient concentrations that occur during the monitoring schedule as well as to programs to reduce nutrient pollution. Additionally, because the WL affects water quality in Lake Poyang, more emphasis should be placed on the low-water period which manifests a relatively bad water quality state, especially due to the prevailing low WL observed recently.

## Materials and Methods

### Study area

**Figure 5:**
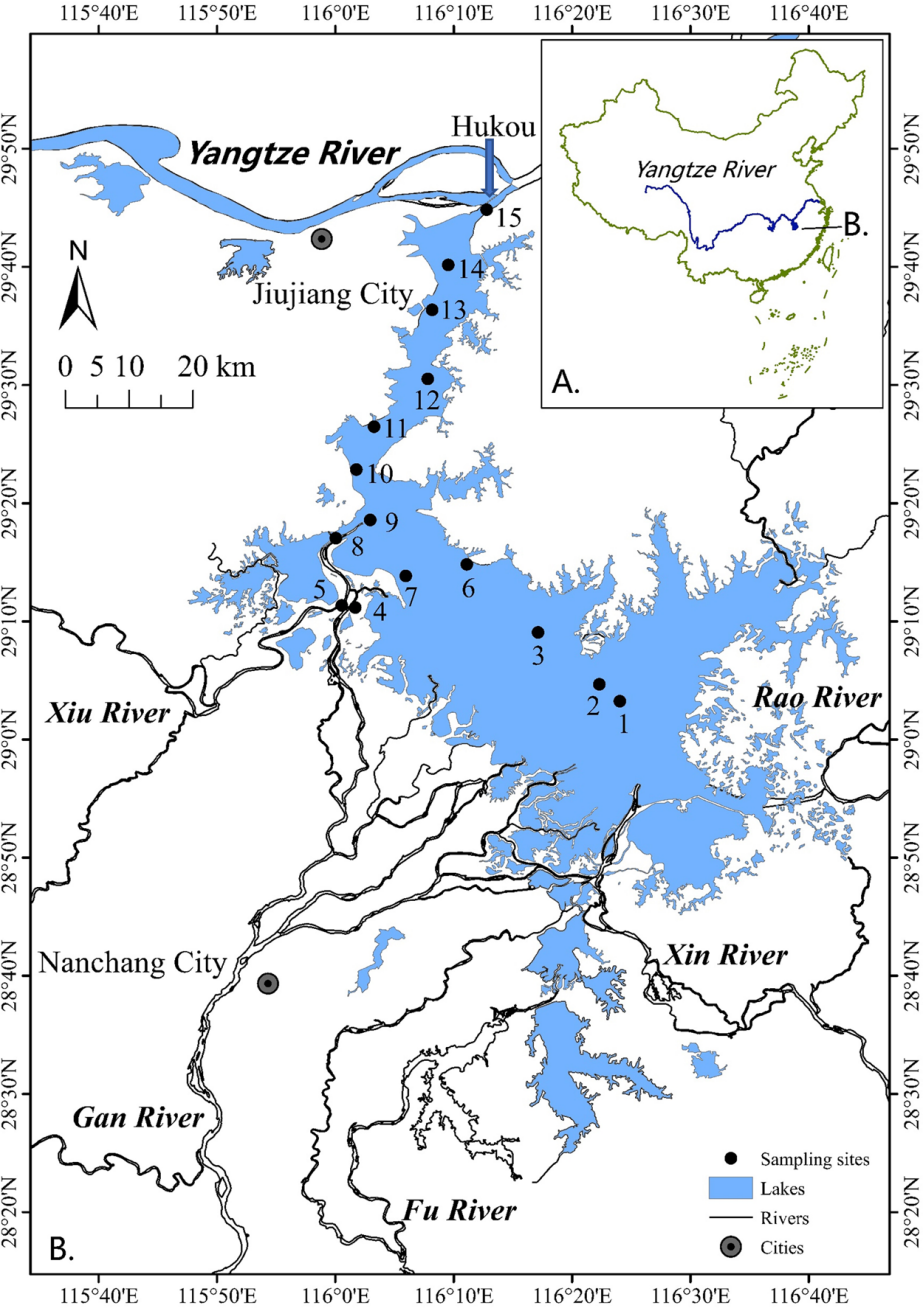
Location of Lake Poyang, China, and the sampling sites. This map was generated in ESRI ArcMap 10.1 (Environmental Systems Resource Institute, ArcMap 10.1 ESRI, Redlands, California, USA, http://www.esri.com/).

Lake Poyang (28°22′-29°45′N, 115°47′-116°45′ E) is located in Jiangxi Province, China, and downstream of the Yangtze River (Fig. 5). With a catchment area of 1.622 × 10^5^ km^2^, it is the largest freshwater lake in China. Approximately 45 million (in 2012) people inhabit the Lake Poyang catchment. The annual discharge from the lake is approximately 1.457 × 10^11^ m^3^, which accounts for 15.6% of the average runoff of the Yangtze River. Additionally, Lake Poyang’s storage capacity is approximately 2.95 × 10^10^ m^3 32^. The average annual rainfall of Lake Poyang ranges from 1340 mm to 1780 mm and occurs primarily in April, May, and June. The evapotranspiration in Lake Poyang ranges from 800 mm–1200 mm; this mainly occurs from July to September^32^. Lake Poyang has five main inflows: the Gan, Fu, Xin, Xiu, and Rao rivers. The mean inflows discharged from the lake’s catchment rivers are 1326 m^3^ s^−1^, 6055 m^3^ s^−1^, 4570 m^3^ s^−1^, and 1563 m^3^ s^−1^, respectively, in January, April, July, and October, during the period of 1956–2008; the corresponding outflow from Lake Poyang to the Yangtze River in Hukou which is located near Site 15 (Fig. 1), was 1799 m^3^ s^−1^, 7017 m^3^ s^−1^, 5980 m^3^ s^−1^, and 3943 m^3^ s^−1^ ^23,53^. Due to its abundant resources (water, sand, wind, etc.), the lake plays a critical role in local economic development. Notably, the local GDP increased by 16.6% from 1998 to 2008^36^.

### Sample collection and laboratory analysis

Sampling was conducted at 15 sampling sites in Lake Poyang four times a year (winter = January; spring = April; summer = July; and autumn = October) from January 2009 to October 2014 (Fig. 5). Selected environmental parameters, including water temperature (T), pH, and dissolved oxygen (DO) were obtained using a multiparametric probe (Hydrolab Datasonde 5 sensor, USA) *in situ*. Because of its connection to the Yangtze River, thermal stratification was weak in Lake Poyang, a finding also reported by Li^54^. The vertically integrated water samples, which were taken at 0.5 m below the surface, from the middle depth, and 0.5 m from the bottom, were collected with acid-cleaned, 10-L plastic buckets. To analyze the heavy metals, 500 mL of the samples were immediately filtered through a 0.45-µm cellulose acetate membrane that had previously been combusted in a Muffle furnace at 500 °C for 5 hours and washed with 0.05 M HNO_3_. The filtrates (50 mL) were collected in acid-washed polypropylene bottles and acidified to a pH of 1 to 2 with ultra-clean HNO_3_. All samples were kept in cold storage and transferred from the field to the laboratory using a cooler filled with ice. The dissolved metals were analyzed in the laboratory within two days.

TN and TP concentrations were measured using a combined persulfate digestion^55^. The concentrations of NH_4_-N, NO_2_-N, NO_3_-N, and PO_4_-P were determined spectrophotometrically (with a UV spectrophotometer (UV 2450)), using the nesslerization, N-(1-naphthyl)-ethylenediamine, phenol disulfonic acid, and molybdenum blue methods, respectively. COD_Mn_ concentrations were determined using a titrimetric method by acid digestion with potassium permanganate oxidation. The concentrations of inorganic anions, such as Cl and SO_4_, were determined using an ion chromatograph. Dissolved metal samples were analyzed directly using a Perkin-Elmer SCIEX Elan 9000 inductively coupled plasma-mass spectrometer (ICP-MS; Model Elan DRC-e; PerkinElmer-SCIEX, CT, USA) equipped with a cross-flow nebulizer (Meinhard Associates, Golden, USA), a Scott-type spray chamber (Glass Expansion, Inc., West Melbourne, Australia), and an AS-93 Plus autosampler (PerkinElmer, Norwalk, CT, USA). The dissolved metals analyzed in this study were As, Hg, Cd, Cr, Pb, Cu, Zn, Fe, Mn, Co, Ni, and V. Additionally, WL data were collected at the Xingzi Hydrology Station during each sampling period.

### WQI calculations

The WQI calculations adopted here were based on the “Environmental Quality Standards for Surface Water” in China, the Environmental Protection Bureau of Guangdong Province (EPBG) and Water Resources Department of Guangdong Province (WRDG)^56^, and Hou^16^. In GB3838-2002, water quality is classified as I, II, III, IV, or V, which correspond to excellent, good, moderate, poor, and bad water states, respectively. The environmental parameters applied in the assessment were divided into three groups: Group 1 (toxic metals), Group 2 (easily treated parameters), and Group 3 (others). The toxic metals in our study included As, Hg, Cd, Cr, and Pb; Group 2 parameters such as pH, DO, TN, TP, NH_4_-N, and COD_Mn_, which can be easily treated by a sewage treatment plant, were classified as easily treated parameters; and Group 3 included Cu, Zn, Fe, Mn, Co, Ni, V, Cl, and SO_4_.

The WQI value of each parameter (I_i_) was calculated as follows:

Where *C*_*i,k*_ ≤ *C*_*i*_ ≤ *C*_*i,k*+*1*_, *I*_*i*_ = $$\frac{{C}_{i}-{C}_{i,k}}{{C}_{i,k+n}-{C}_{i,k}}\times 20n+{I}_{i,k}$$; *C*_*i*_ ≤ *C*_*i*, *1*_, *I*_*i*=_0; and *C*_*i,5*_ ≤ *C*_*i*_, *I*_*i*=_100.

In the formula above, *C*_*i*_ is the concentration of the *i* ^*th*^ parameter in the evaluation; *C*_*i*,*k*_ and *C*_*i*,*k*+1_ are the normal concentrations of the *i* ^*th*^ parameters of class *k* and class *k* + *1*, respectively; *I*_*i*_ is the WQI value of the *i* ^*th*^ parameter; *n* is the number of the same standard value for the *i* ^*th*^ parameter in GB3838–2002; *I*_*i*,*k*_ is the corresponding value for class K; and the value of classes I, II, III, IV, and V are 20, 40, 60, 80, and 100, respectively. The detailed normal concentrations of the environmental parameters included in our study can be found in the supplementary material (Table S2).

Notably, some parameters, such as Fe, Mn, Co, Ni, V, Cl, and SO_4_, have only one normal concentration for all five classes. Thus, for these parameters, the WQI was expressed as follows: ${\rm{WQI}}=\frac{{C}_{i}}{{C}_{i,k}}\times 60$. In Lake Poyang, the water quality goal is class III or higher, and *C*_*i*,*k*_ represents the normal concentration of the *i* ^*th*^ parameter corresponding to class III. Additionally, *I* = 0 when 6 ≤ pH ≤ 9; otherwise, *I* = 100.

The WQI calculation is different for each group based on I_i_. The WQI for toxic metals, i.e, W(1), was calculated as W(1) = max(I_i_). For easily treated and other parameters, the WQI values were the mean values of I_i_, i.e., ${\rm{WQI}}=\frac{1}{n}\sum _{i=1}^{n}{I}_{i}$. Finally, the WQI for all three groups was calculated as WQI = max (W(1, 2, 3)). The maximum and minimum WQI values were 100 and 0, respectively, where higher values represent poorer water quality conditions. The water quality was classified into five grades based on the WQI scores: excellent (0–20), good (21–40), moderate (41–60), low (61–80), and bad (81–100).

### Data analysis

All data were log (x + 1)-transformed prior to analysis to meet the conditions of normality and homogeneity of variance in the residuals. Pearson’s correlation analyses were performed using the SPSS statistical package for Windows (version 17.0) to detect relationships between water quality parameters and the WQI. To analyze the effect of WL on WQI, we averaged the WL and WQI data for each sampling period and obtained 24 values for both parameters. To detect the effect of WL on WQI on an interannual scale, the data were averaged annually for all four seasons to eliminate seasonal influences.

### Data Availability

The datasets generated and analysed during the current study are not publicly available due to further analysis for other purposes but are available from the corresponding authors on reasonable request.

## Electronic supplementary material

Supplementary Information

## References

[1] John V, Jain P, Rahate M, Labhasetwar P (2014). Assessment of deterioration in water quality from source to household storage in semi-urban settings of developing countries. Environ. Monit. Assess..

[2] Scanlon BR, Jolly I, Sophocleous M, Zhang L (2007). Global impacts of conversions from natural to agricultural ecosystems on water resources: Quantity versus quality. Water Resour. Res..

[3] Todd AS (2012). Climate-change-driven deterioration of water quality in a mineralized watershed. Environ. Sci. Technol..

[4] UNICEF & WHO. *Meeting The MDG Drinking Water And Sanitation Target: A Mid-term Assessment Of Progress*. (WHO Press: Geneva, Switzerland (2004).

[5] Astel A, Biziuk M, Przyjazny A, Namiesnik J (2006). Chemometrics in monitoring spatial and temporal variations in drinking water quality. Water Res..

[6] Behmel S, Damour M, Ludwig R, Rodriguez MJ (2016). Water quality monitoring strategies - A review and future perspectives. Sci. Total Environ..

[7] Romero E (2016). Long-term water quality in the lower Seine: Lessons learned over 4 decades of monitoring. Environ. Sci. Policy.

[8] Singh KP, Malik A, Sinha S (2005). Water quality assessment and apportionment of pollution sources of Gomti river (India) using multivariate statistical techniques - a case study. Anal. Chim. Acta.

[9] Huang JC, Gao JF, Zhang YJ (2016). Eutrophication Prediction Using a Markov Chain Model: Application to Lakes in the Yangtze River Basin, China. Environ. Model. Assess..

[10] Wu NC, Schmalz B, Fohrer N (2012). Development and testing of a phytoplankton index of biotic integrity (P-IBI) for a German lowland river. Ecol. Indic..

[11] Horton RK (1965). An index number system for rating water quality. J. Water Pollut. Control Fed..

[12] Brown RM, McClelland NI, Deininger RA, Tozer RG (1970). A water quality index - do we dare?. Water Sew. Works.

[13] Debels P, Figueroa R, Urrutia R, Barra R, Niell X (2005). Evaluation of water quality in the Chilla’n River (Central Chile) using physicochemical parameters and a modified Water Quality Index. Environ. Monit. Assess..

[14] Wu ZS, Wang XL, Chen YW, Cai YJ, Deng JC (2018). Assessing river water quality using water quality index in Lake Taihu Basin, China. Sci. Total Environ..

[15] Wang, X. P., Zhang, F. & Ding, J. L. Evaluation of water quality based on a machine learning algorithm and water quality index for the Ebinur Lake Watershed, China. *Sci*. *Rep*. **7** (2017). 10.1038/s41598-017-12853-y PMC5634425 28993639

[16] Hou W (2016). Assessing water quality of five typical reservoirs in lower reaches of Yellow River, China: Using a water quality index method. Ecol. Indic..

[17] Sener S, Sener E, Davraz A (2017). Evaluation of water quality using water quality index (WQI) method and GIS in Aksu River (SW-Turkey). Sci. Total Environ..

[18] Selvam S, Manimaran G, Sivasubramanian P, Balasubramanian N, Seshunarayana T (2014). GIS-based Evaluation of Water Quality Index of groundwater resources around Tuticorin coastal city, south India. Environ. Earth Sci..

[19] Jin XC, Xu QJ, Huang CZ (2005). Current status and future tendency of lake eutrophication in China. Sci. China.Series C: Life Sci..

[20] Le C (2010). Eutrophication of Lake Waters in China: Cost, Causes, and Control. Environ. Manage..

[21] Yang GS (2010). Lake status, major problems and protection strategy in China. J. Lake Sci..

[22] Wu ZS, He H, Cai YJ, Zhang L, Chen YW (2014). Spatial distribution of chlorophyll a and its relationship with the environment during summer in Lake Poyang: a Yangtze-connected lake. Hydrobiol..

[23] Wu, Z., Lai, X., Zhang, L., Cai, Y. & Chen, Y. Phytoplankton chlorophyll a in Lake Poyang and its tributaries during dry, mid-dry and wet seasons: a 4-year study. *Knowl*. *Manag*. *Aquat*. *Ecosyst*. 1–13 (2014).

[24] Wu Z (2013). Temporal and spatial variability of phytoplankton in Lake Poyang: The largest freshwater lake in China. J. Great Lakes Res..

[25] Zhang D (2012). Distribution of heavy metals in water, suspended particulate matter and sediment of Poyang Lake, China. Fresen. Environ. Bull..

[26] Duan WL (2016). Water Quality Assessment and Pollution Source Identification of the Eastern Poyang Lake Basin Using Multivariate Statistical Methods. Sustainability.

[27] Dou, H. S. & Jiang, J. H. *The Five Freshwater Lake In China*. (Press of University of Science & Technology of China:Hefei, China, 2003).

[28] Lv LJ (1992). Water quality condition analysis and evaluation in Lake Poyang. Yangtze River.

[29] Qian Y, Migliaccio KW, Wan YS, Li YC (2007). Surface water quality evaluation using multivariate methods and a new water quality index in the Indian River Lagoon, Florida. Water Resour. Res..

[30] Beklioglu, M., Altinayar, G. & Tan, C. O. Role of water level fluctuations, nutrients and fish in determining the macrophyte dominated clear water states in five Turkish shallow lakes. Shallow Lake Wetlands: Ecology, Eutrophication and Restoration. International Workshop, 28–30 October 2001, Ankara, Turkey (2001).

[31] Coops H, Beklioglu M, Crisman TL (2003). The role of water-level fluctuations in shallow lake ecosystems - workshop conclusions. Hydrobiol..

[32] Zhu, H. H. & Zhang, B. The Lake Poyang. (University of Science & Technology of China Press: Hefei, China, 1997).

[33] Shankman D, Keim BD, Song J (2006). Flood frequency in China’s Poyang Lake region: Trends and teleconnections. Int. J. Climatol..

[34] Lai X (2014). Sand mining and increasing Poyang Lake’s discharge ability: A reassessment of causes for lake decline in China. J. Hydrol..

[35] Zhang Q (2014). An investigation of enhanced recessions in Poyang Lake: Comparison of Yangtze River and local catchment impacts. J. Hydrol..

[36] Wang, S. R. *Water Environment In Lake Poyang*. (Science Press: Beijing, China, 2014).

[37] Liu FG, Li M, Guo YY (2014). Spatial-temporal variation of water quality and water level effect on water quality in Poyang Lake. J. China Hydrol..

[38] Bordalo AA, Teixeira R, Wiebe WJ (2006). A water quality index applied to an international shared river basin: The case of the douro river. Environ. Manage..

[39] Lv LJ (1996). Investigation on Poyang Lake water pollution by eutrophication. J. Lake Sci..

[40] Zhang YH, Yang GS, Li B, Cai YJ, Chen YW (2016). Using eutrophication and ecological indicators to assess ecosystem condition in Poyang Lake, a Yangtze-connected lake. Aquat. Ecosystem Health Manage..

[41] Howarth RW, Sharpley A, Walker D (2002). Sources of nutrient pollution to coastal waters in the United States: Implications for achieving coastal water quality goals. Estuaries.

[42] Nielsen A (2014). Effects of climate and nutrient load on the water quality of shallow lakes assessed through ensemble runs by PCLake. Ecol. Appl..

[43] Waltham, N. J., Reichelt-Brushett, A., McCann, D. & Eyre, B. D. Water and sediment quality, nutrient biochemistry and pollution loads in an urban freshwater lake: balancing human and ecological services. *Environ*. *Sci*. *Proc*. *Impacts* **16**, 2804–2813 (2014). 10.1039/c4em00243a 25384753

[44] Evtimova VV, Donohue I (2016). Water-level fluctuations regulate the structure and functioning of natural lakes. Freshwater Bio..

[45] Liu X, Teubner K, Chen YW (2016). Water quality characteristics of Poyang Lake, China, in response to changes in the water level. Hydrol. Res..

[46] Wang X, Xiao WH, Zhu WY, Shi X (2012). Effects of water level variation on water quality in Dongting Lake. South-to-North Water Transfers and Water Science & Technology.

[47] Wang YY (2011). Potential influence of water level changes on energy flows in a lake food web. Chin. Sci. Bull..

[48] Sondergaard M, Kristensen P, Jeppesen E (1992). Phosphorus release from resuspended sediment in the shallow and wind-exposed Lake Arreso, Denmark. Hydrobiol..

[49] Hakanson L, Parparov A, Hambright KD (2000). Modelling the impact of water level fluctuations on water quality (suspended particulate matter) in Lake Kinneret, Israel. Ecol. Modell..

[50] Gianniou SK, Antonopoulos VZ (2007). Evaporation and energy budget in lake vegoritis, Greece. J. Hydrol..

[51] Stefanidis K, Papastergiadou E (2013). Effects of a long term water level reduction on the ecology and water quality in an eastern Mediterranean lake. Knowl. Manag. Aquat. Ecosyst..

[52] Naselli-Flores L, Barone R (2005). Water-level fluctuations in Mediterranean reservoirs: setting a dewatering threshold as a management tool to improve water quality. Hydrobiol..

[53] Lai XJ, Huang Q, Zhang YH, Jiang JH (2014). Impact of lake inflow and the Yangtze River flow alterations on water levels in Poyang Lake, China. Lake Reserv. Manag..

[54] Li YL, Yao J, Zhang L (2016). Investigation into mixing in the shallow floodplain Poyang Lake (China) using hydrological, thermal and isotopic evidence. Water Sci. Technol..

[55] Ebina J, Tsutsui T, Shirai T (1983). Simultaneous determination of total nitrogen and total phosphorus in water using peroxodisulfate oxidation. Water Res..

[56] Environmental Protection Bureau of Guangdong Province (EPBG) & Water Resources Department of Guangdong Province (WRDG). Water quality monitoring, evaluation, and scheme of water resource in Guangdong province cities’ drinking water (2002).

