# Supplementary material for: Water quality assessment based on the water quality index method in Lake Poyang: The largest freshwater lake in China
Source: Sci Rep. 2017 Dec 21;7:17999. doi: 10.1038/s41598-017-18285-y (PMC5740168; doi:10.1038/s41598-017-18285-y)
Supplement: Supplementary file 1 — Supplementary Information [file 41598_2017_18285_MOESM1_ESM.doc]

Supplementary information

**Water quality assessment based on the water quality index method in Lake Poyang: The largest freshwater lake in China**

Zhaoshi Wu^1^,*, Dawen Zhang^2^, Yongjiu Cai^1^, Xiaolong Wang^1^, Lu Zhang^1^, Yuwei Chen^1^,*

^1^State Key Laboratory of Lake Science and Environment, Nanjing Institute of Geography and Limnology, Chinese Academy of Sciences, 73 East Beijing Road, Nanjing 210008, China

^2^Institute for Quality & Safety and Standards of Agricultural Products Research,
Jiangxi Academy of Agricultural Sciences, Nanchang, 330200, P.R. China

Emails: [zswu@niglas.ac.cn](mailto:zswu@niglas.ac.cn); [ywchen@niglas.ac.cn](mailto:ywchen@niglas.ac.cn)

## Table S1 Water quality parameters summarized as mean values and ranges at 15 sites in Lake Poyang, China, from 2009 to 2014 (mg L^-1^ excluded pH).

| Group | Variable | Abbreviation | Mean | Minimum | Maximum |
| --- | --- | --- | --- | --- | --- |
| 1. toxic metals | Arsenic | As | 0.0028 | 0.0002 | 0.0575 |
|  | Mercury | Hg | 0.00001 | 0.0000 | 0.0002 |
|  | Cadmium | Cd | 0.0002 | 0.0000 | 0.0039 |
|  | Chromium | Cr | 0.0012 | 0.0000 | 0.0528 |
|  | Lead | Pb | 0.0035 | 0.0000 | 0.0466 |
| 2. easily treated  parameters | pH | pH | 8.1267 | 6.5300 | 11.6500 |
|  | Dissolved oxygen | DO | 8.7929 | 4.3000 | 15.9000 |
|  | Total nitrogen | TN | 1.7988 | 0.3970 | 8.7460 |
|  | Total phosphorus | TP | 0.0979 | 0.0170 | 0.4600 |
|  | Ammonium | NH_4_-N | 0.3843 | 0.2431 | 0.6275 |
|  | Permanganate index | COD_Mn_ | 1.3556 | 2.4222 | 3.7778 |
| 3. other parameters | Copper | Cu | 0.0054 | 0.0000 | 0.0960 |
|  | Zinc | Zn | 0.1764 | 0.0007 | 11.7697 |
|  | Iron | Fe | 0.0364 | 0.0000 | 0.2941 |
|  | Manganese | Mn | 0.0363 | 0.0001 | 0.9916 |
|  | Cobalt | Co | 0.0001 | 0.0000 | 0.0004 |
|  | Nickel | Ni | 0.0046 | 0.0001 | 0.1331 |
|  | Vanadium | V | 0.0010 | 0.0000 | 0.0040 |
|  | Chloride | Cl | 20.4683 | 4.0400 | 150.0108 |
|  | Sulphate | SO_4_ | 18.4394 | 6.5300 | 73.4548 |

## Table S2 Normal concentrations of the parameters used in the calculation of the water quality index (mg L^-1^ excluded pH).

| Group | Variables | Normal concentration | | | | | |
| --- | --- | --- | --- | --- | --- | --- | --- |
|  |  | I | II | III | IV | V |  |
|  |  | *I_i,k_*_=20_ | *I_i,k_*_=40_ | *I_i,k_*_=60_ | *I_i,k_*_=80_ | *I_i,k_*_=100_ |  |
| 1. toxic metals | As | ≤0.05 | ≤0.05 | ≤0.05 | ≤0.1 | ≤0.1 |  |
|  | Hg | ≤0.00005 | ≤0.00005 | ≤0.0001 | ≤0.001 | ≤0.001 |  |
|  | Cd | ≤0.001 | ≤0.005 | ≤0.005 | ≤0.005 | ≤0.01 |  |
|  | Cr | ≤0.01 | ≤0.05 | ≤0.05 | ≤0.05 | ≤0.1 |  |
|  | Pb | ≤0.01 | ≤0.01 | ≤0.05 | ≤0.05 | ≤0.1 |  |
| 2. easily treated  parameters | pH | 6/9 | 6/9 | 6/9 | 6/9 | 6/9 |  |
|  | DO | ≥7.5 | ≥6 | ≥5 | ≥3 | ≥2 |  |
|  | TN | ≤0.2 | ≤0.5 | ≤1.0 | ≤1.5 | ≤2.0 |  |
|  | TP | ≤0.01 | ≤0.025 | ≤0.05 | ≤0.1 | ≤0.2 |  |
|  | NH_4_-N | ≤0.15 | ≤0.5 | ≤1.0 | ≤1.5 | ≤2.0 |  |
|  | COD_Mn_ | ≤2 | ≤4 | ≤6 | ≤10 | ≤15 |  |
| 3.other parameters | Cu | ≤0.01 | ≤1.0 | ≤1.0 | ≤1.0 | ≤1.0 |  |
|  | Zn | ≤0.05 | ≤1.0 | ≤1.0 | ≤2.0 | ≤2.0 |  |
|  | Fe | ≤0.3 | ≤0.3 | ≤0.3 | ≤0.3 | ≤0.3 |  |
|  | Mn | ≤0.1 | ≤0.1 | ≤0.1 | ≤0.1 | ≤0.1 |  |
|  | Co | ≤1.0 | ≤1.0 | ≤1.0 | ≤1.0 | ≤1.0 |  |
|  | Ni | ≤0.02 | ≤0.02 | ≤0.02 | ≤0.02 | ≤0.02 |  |
|  | V | ≤0.05 | ≤0.05 | ≤0.05 | ≤0.05 | ≤0.05 |  |
|  | Cl | ≤250 | ≤250 | ≤250 | ≤250 | ≤250 |  |
|  | SO_4_ | ≤250 | ≤250 | ≤250 | ≤250 | ≤250 |  |

Adopted from the surface water quality standard GB3838-2002 (China).
